# Supplementary figures and images for: The robust, high-throughput, and temporally regulated roxCre and loxCre reporting systems for genetic modifications in vivo
Source: eLife. 2026 Apr 20;13:RP97717. doi: 10.7554/eLife.97717 (PMC13095210; doi:10.7554/eLife.97717)

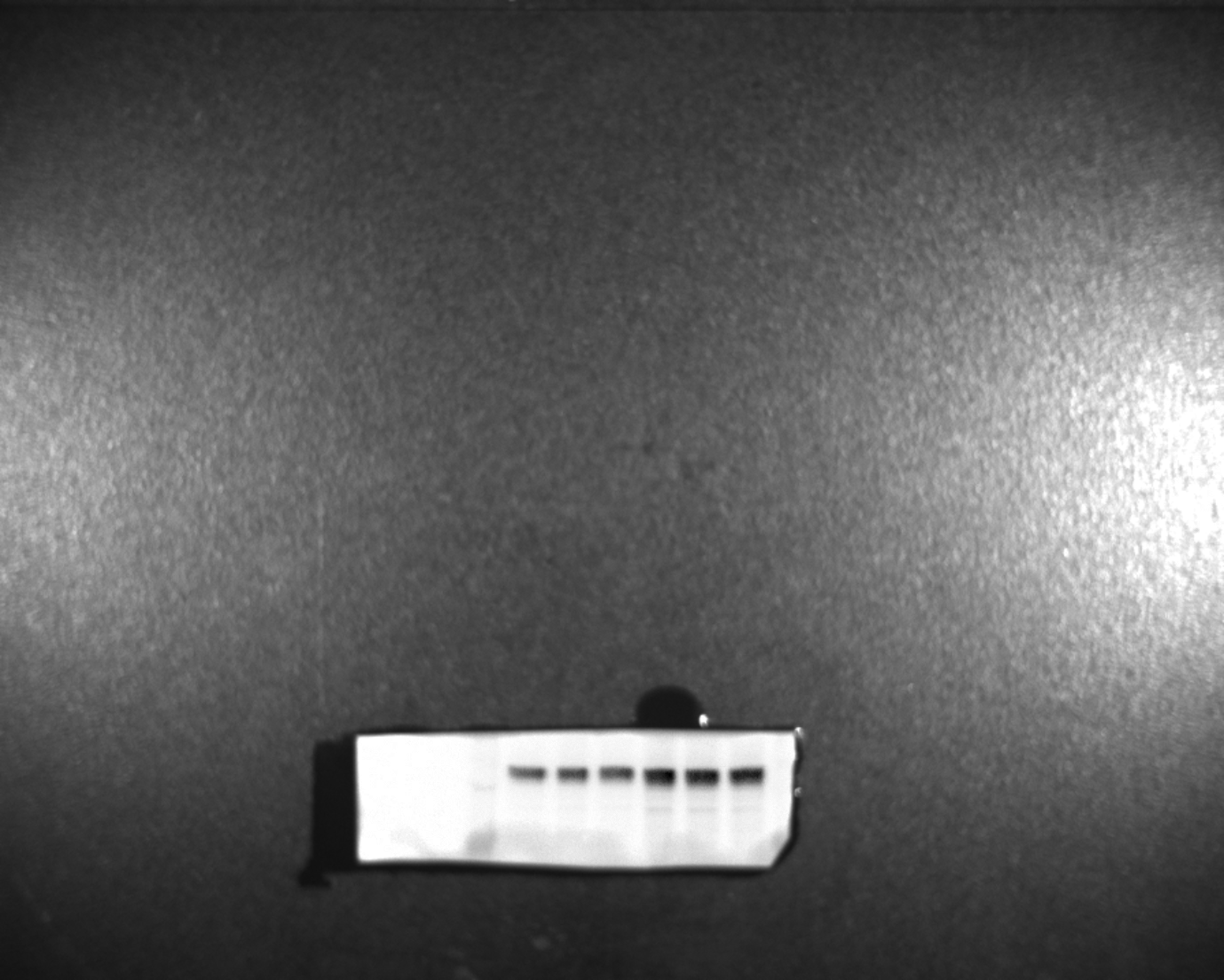

Supplement: Figure 3—source data 2. [file elife-97717-fig3-data2.zip › Figure3-source data 2-Original files for western blot analysis displayed in Figure 3I/Original files for western blot analysis displayed in Figure 3I 2.tif]

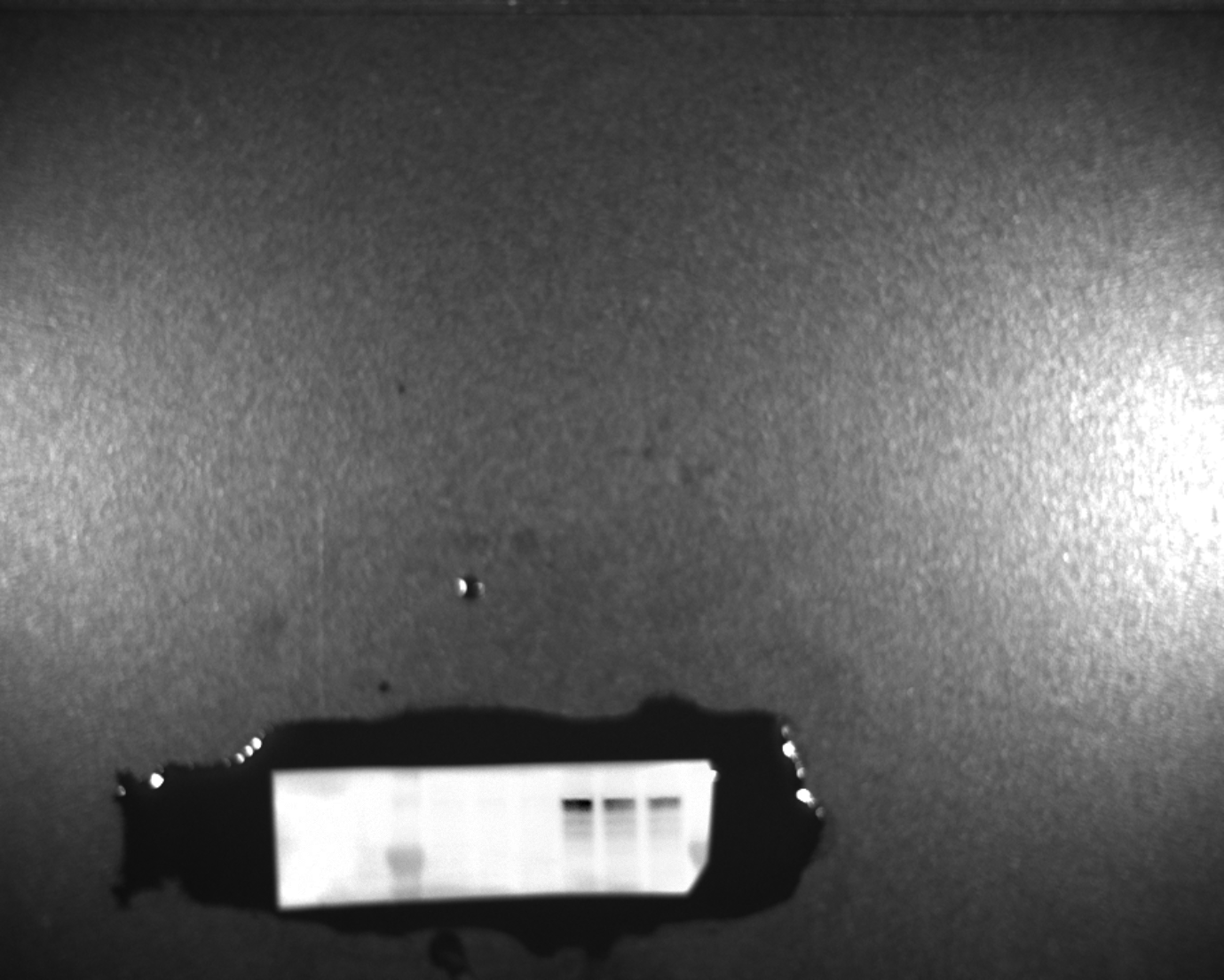

Supplement: Figure 3—source data 2. [file elife-97717-fig3-data2.zip › Figure3-source data 2-Original files for western blot analysis displayed in Figure 3I/Original files for western blot analysis displayed in Figure 3I 1.tif]

Marker(kDa)

50 —  
40 —  
35 —

3#

2#

1#

3#

2#

1#

*Cyp2e1-DreER; Alb-rxCre-tdT; Ctnnb1<sup>fl/fl</sup>*

*Cyp2e1-DreER; Alb-rxCre-tdT; Ctnnb1<sup>fl/+</sup>*

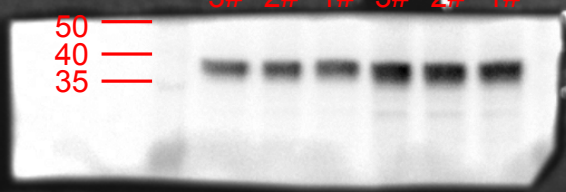

Supplement: Figure 3—source data 3. [file elife-97717-fig3-data3.zip › Figure3-source data 3ΓÇöPDF files containing originall western blots for Figure 3I,indicating the relevant bands and treatments. copy/Source data Figure 3I uncropped western blots 2.pdf]

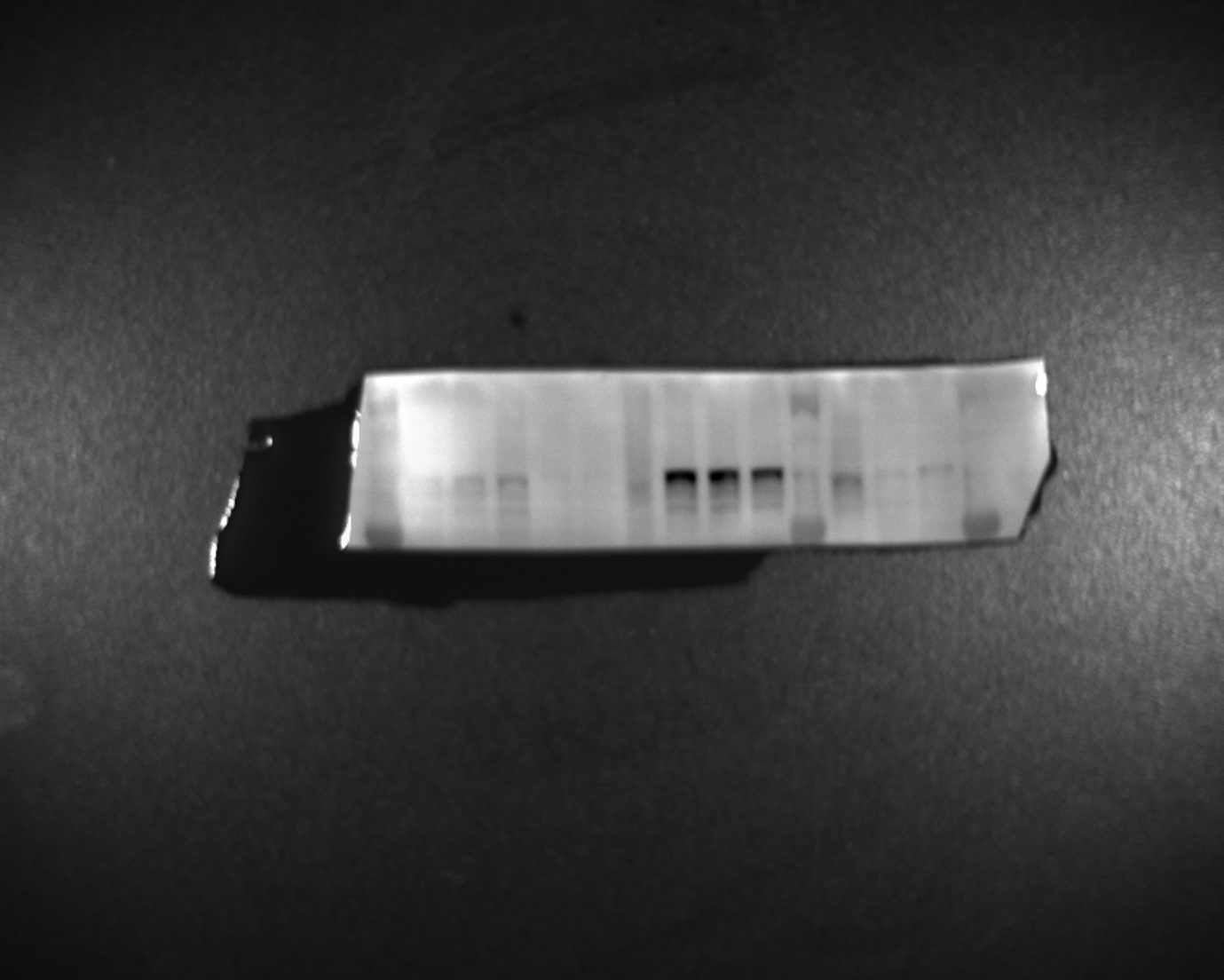

Supplement: Figure 7—source data 2. [file elife-97717-fig7-data2.zip › Figure7-source data 2-Original files for western blot analysis displayed in Figure 7D/Original files for western blot analysis displayed in Figure 7D Beta-catenin.tif]

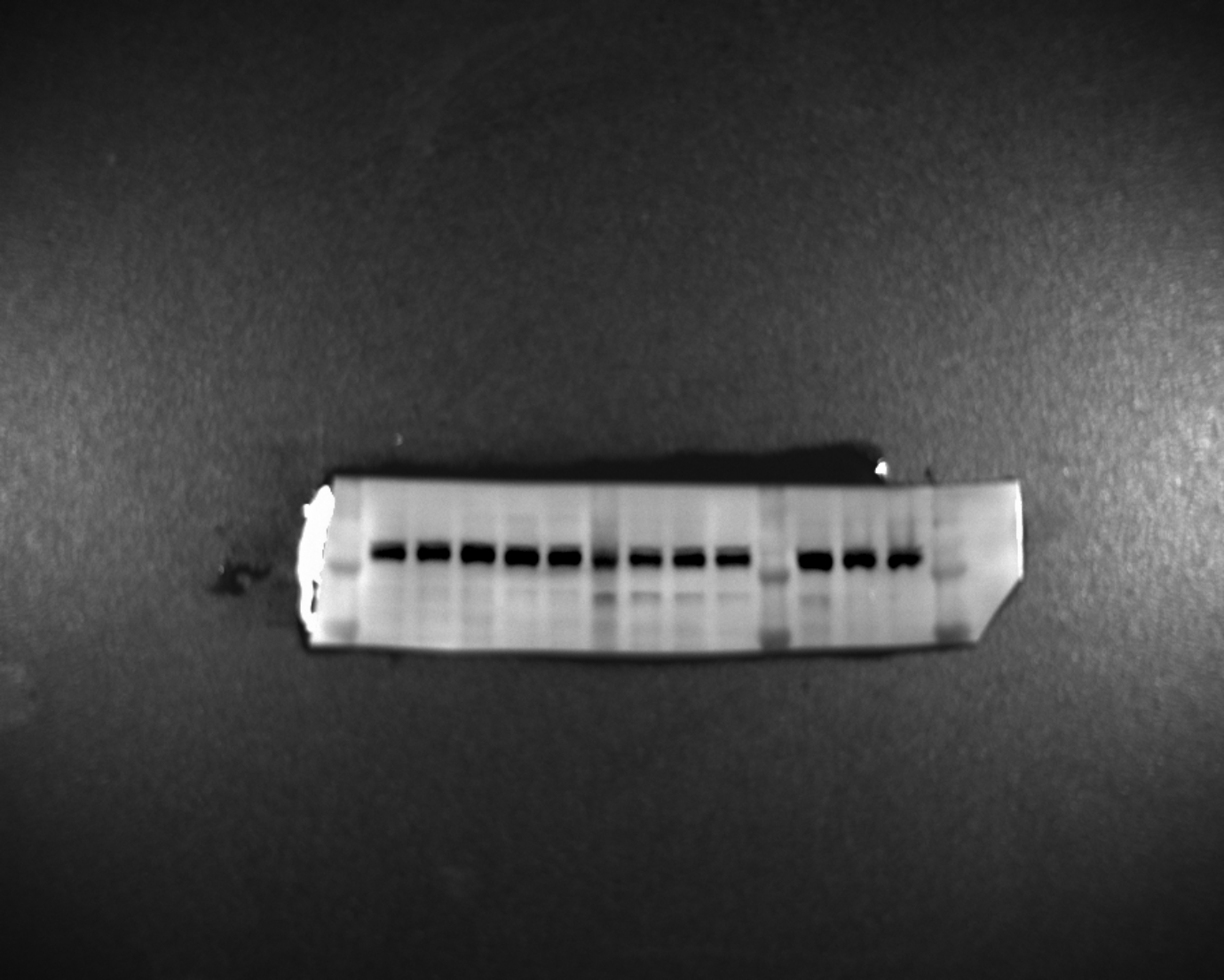

Supplement: Figure 7—source data 2. [file elife-97717-fig7-data2.zip › Figure7-source data 2-Original files for western blot analysis displayed in Figure 7D/Original files for western blot analysis displayed in Figure 7D Beta-actin.tif]

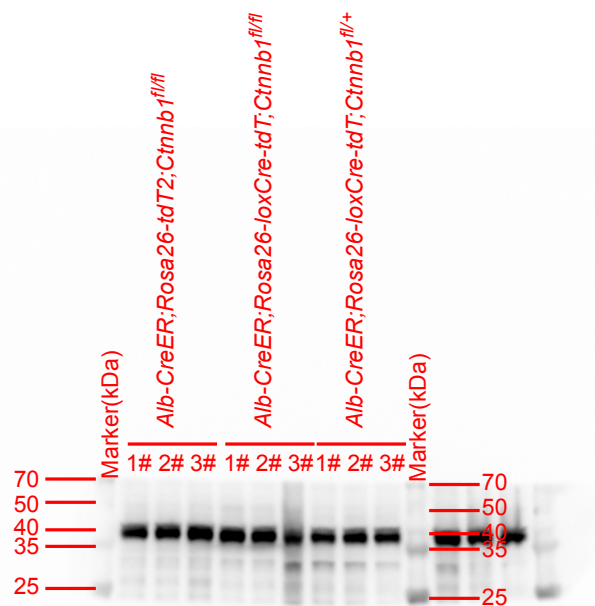

Supplement: Figure 7—source data 3. [file elife-97717-fig7-data3.zip › Figure7-source data 3ΓÇöPDF files containing originall western blots for Figure 7D,indicating the relevant bands and treatments./Source data Figure 7D uncropped western blots 2.pdf]

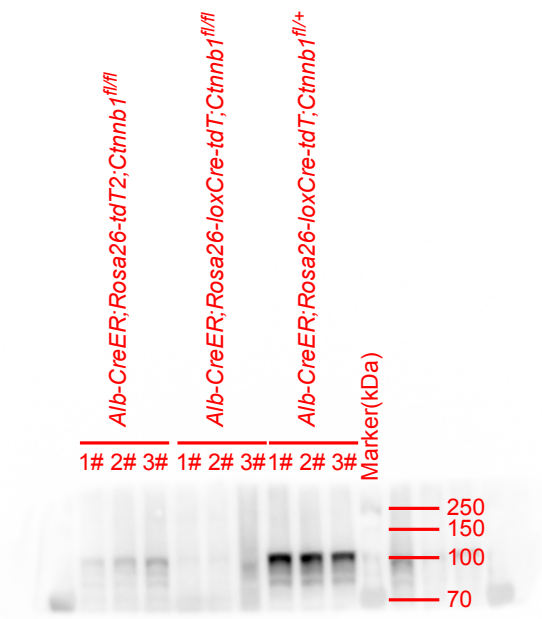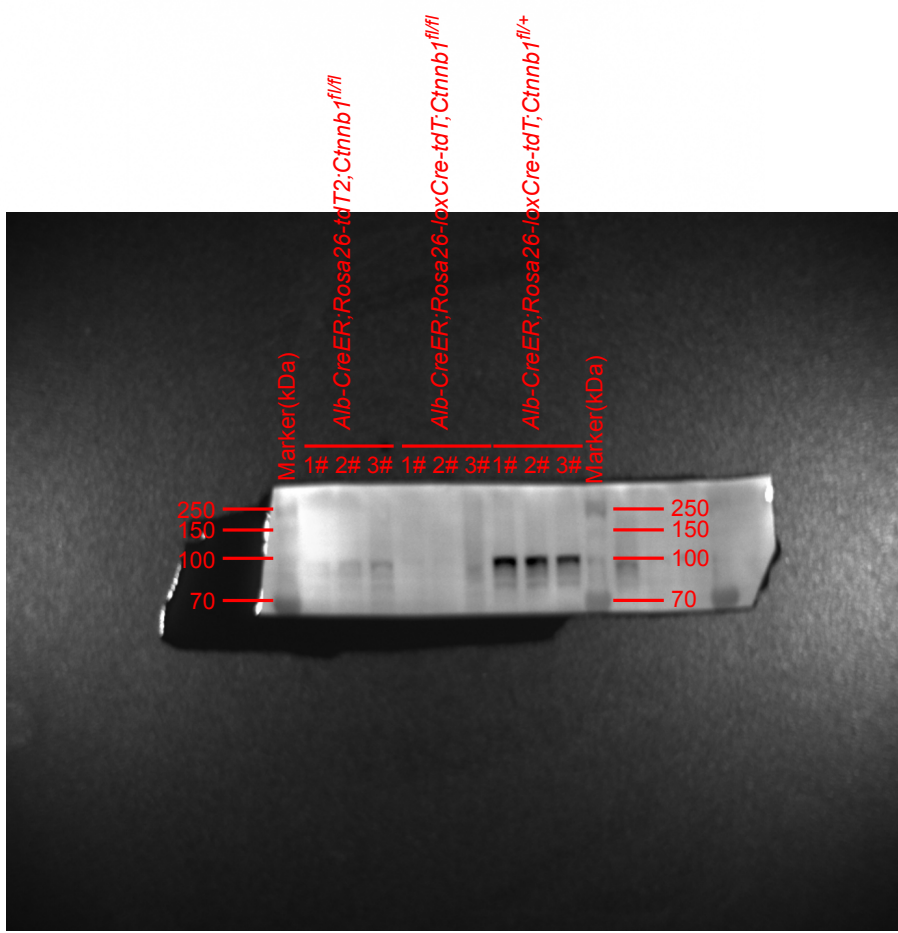

Supplement: Figure 7—source data 3. [file elife-97717-fig7-data3.zip › Figure7-source data 3ΓÇöPDF files containing originall western blots for Figure 7D,indicating the relevant bands and treatments./Source data Figure 7D uncropped western blots 1.pdf]

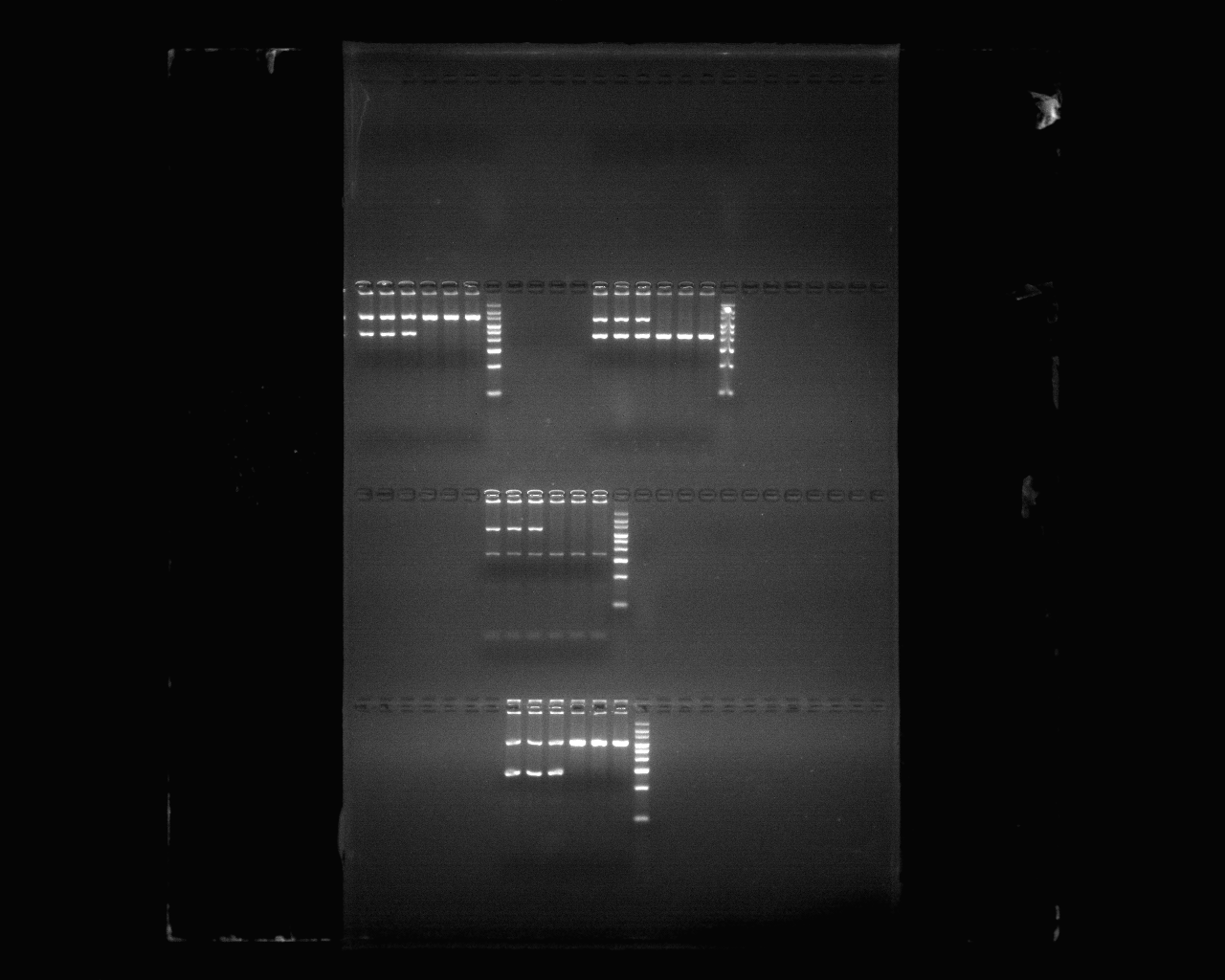

Supplement: Figure 7—figure supplement 2—source data 1. [file elife-97717-fig7-figsupp2-data1.zip › Figure 7ΓÇö figure supplement 2ΓÇösource data 1/The original files for the agarose gel electrophoresis results are shown in Figure 7ΓÇö figure supplement 2_ Alb-roxCre1-tdT and Cdh5-roxCre4-tdT.Tif]

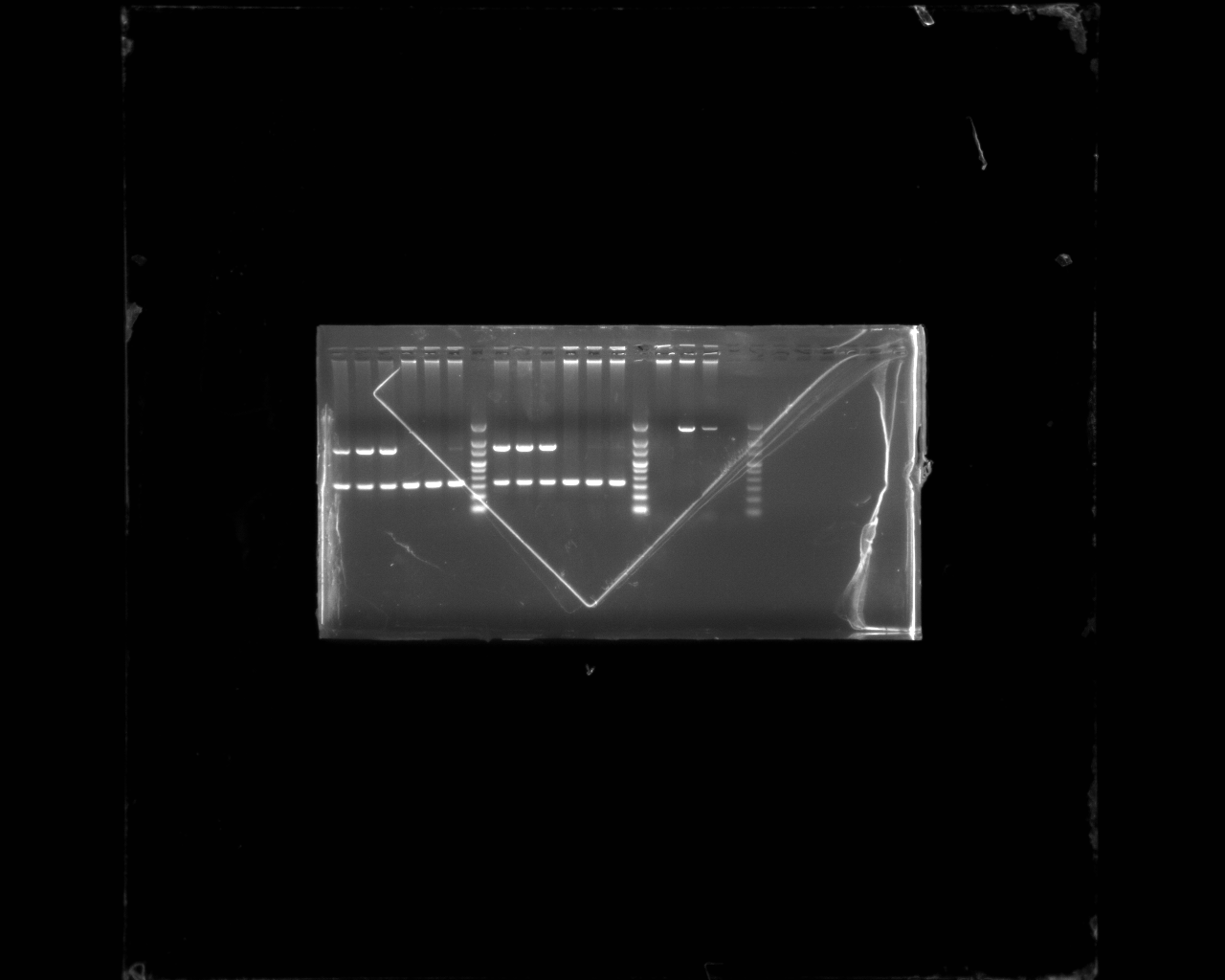

Supplement: Figure 7—figure supplement 2—source data 1. [file elife-97717-fig7-figsupp2-data1.zip › Figure 7ΓÇö figure supplement 2ΓÇösource data 1/The original files for the agarose gel electrophoresis results are shown in Figure 7ΓÇö figure supplement 2_Cdh5-roxCre10-GFP.Tif]

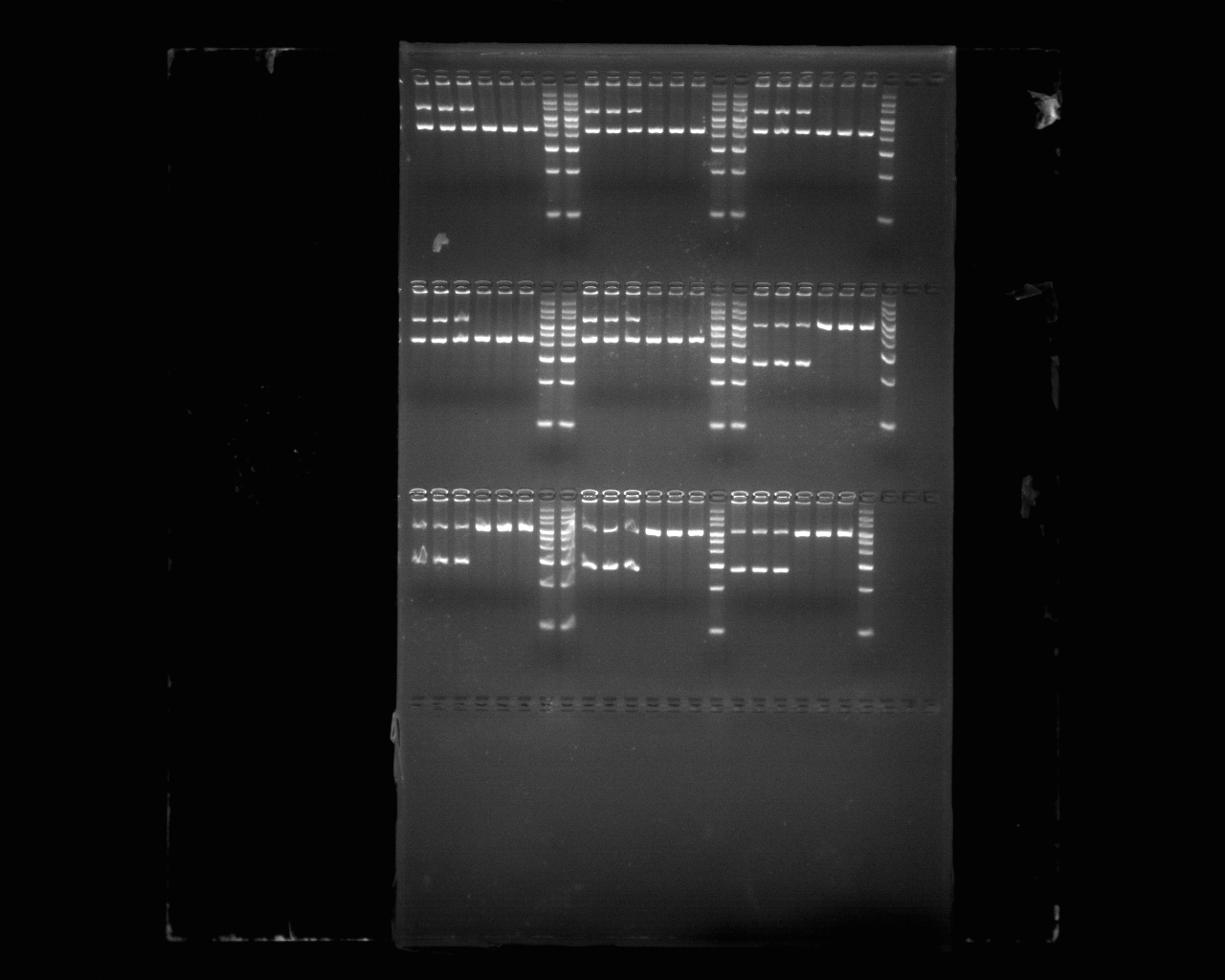

Supplement: Figure 7—figure supplement 2—source data 1. [file elife-97717-fig7-figsupp2-data1.zip › Figure 7ΓÇö figure supplement 2ΓÇösource data 1/The original files for the agarose gel electrophoresis results are shown in Figure 7ΓÇö figure supplement 2_ Alb-roxCre7-GFP.Tif]

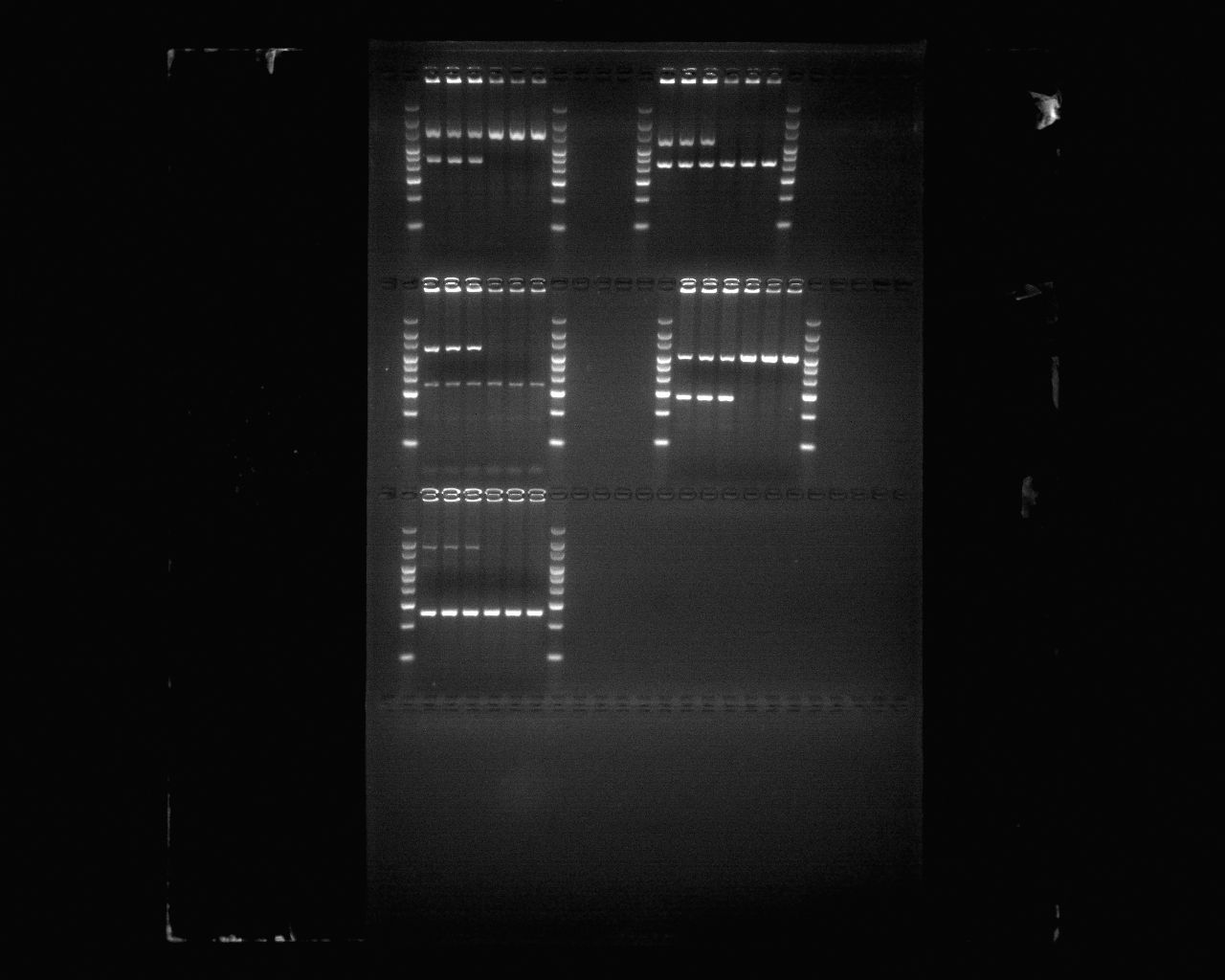

Supplement: Figure 7—figure supplement 2—source data 1. [file elife-97717-fig7-figsupp2-data1.zip › Figure 7ΓÇö figure supplement 2ΓÇösource data 1/The original files for the agarose gel electrophoresis results are shown in Figure 7ΓÇö figure supplement 2_Cyp2e1-DreER, Rosa26-loxCre-tdT .Tif]

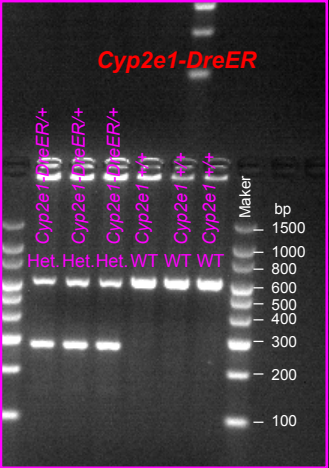

Supplement: Figure 7—figure supplement 2—source data 2. [file elife-97717-fig7-figsupp2-data2.zip › Figure 7ΓÇö figure supplement 2ΓÇösource data 2/Source data Figure 7ΓÇö figure supplement 2_ Cyp2e1-DreER's the agarose gel electrophoresis results.pdf]

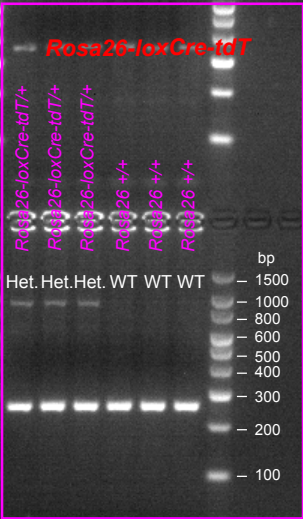

Supplement: Figure 7—figure supplement 2—source data 2. [file elife-97717-fig7-figsupp2-data2.zip › Figure 7ΓÇö figure supplement 2ΓÇösource data 2/Source data Figure 7ΓÇö figure supplement 2_ Rosa26-loxCre-tdT's the agarose gel electrophoresis results.pdf]

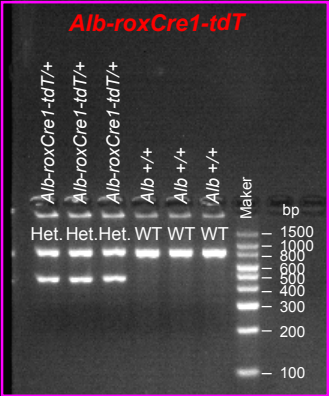

Supplement: Figure 7—figure supplement 2—source data 2. [file elife-97717-fig7-figsupp2-data2.zip › Figure 7ΓÇö figure supplement 2ΓÇösource data 2/Source data Figure 7ΓÇö figure supplement 2_ Alb-roxCre1-tdT's the agarose gel electrophoresis results.pdf]

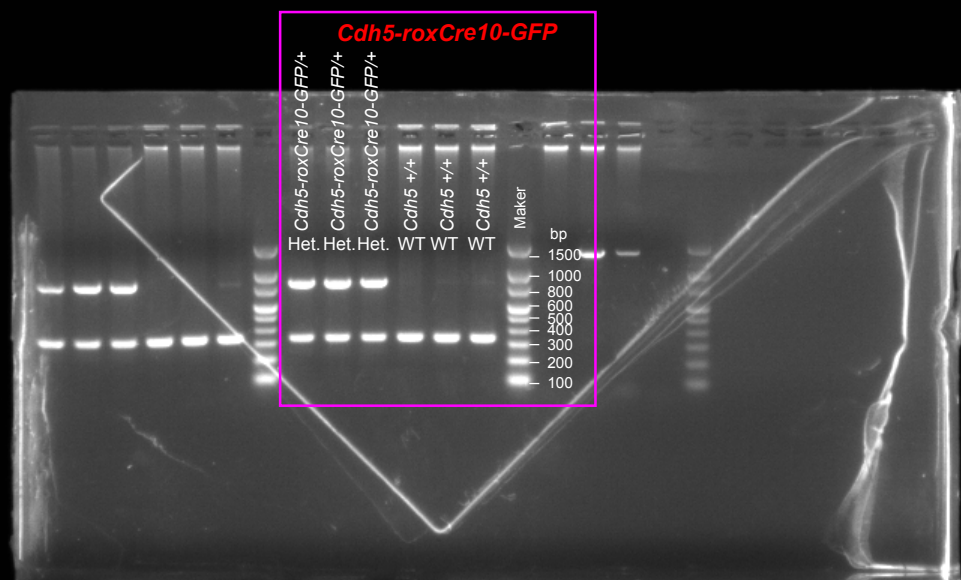

Supplement: Figure 7—figure supplement 2—source data 2. [file elife-97717-fig7-figsupp2-data2.zip › Figure 7ΓÇö figure supplement 2ΓÇösource data 2/Source data Figure 7ΓÇö figure supplement 2_ Cdh5-roxCre10-GFP's the agarose gel electrophoresis results.pdf]

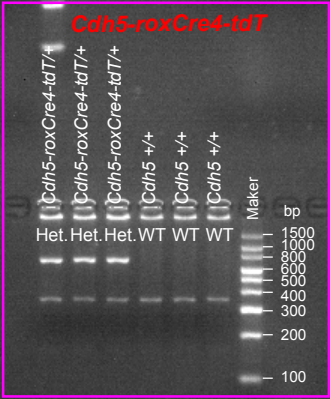

Supplement: Figure 7—figure supplement 2—source data 2. [file elife-97717-fig7-figsupp2-data2.zip › Figure 7ΓÇö figure supplement 2ΓÇösource data 2/Source data Figure 7ΓÇö figure supplement 2_ Cdh5-roxCre4-tdT's the agarose gel electrophoresis results.pdf]
