# Supplementary material for: The robust, high-throughput, and temporally regulated roxCre and loxCre reporting systems for genetic modifications in vivo
Source: eLife. 2026 Apr 20;13:RP97717. doi: 10.7554/eLife.97717 (PMC13095210; doi:10.7554/eLife.97717)
Supplement: Figure 3—source data 3. [file elife-97717-fig3-data3.zip › Figure3-source data 3ΓÇöPDF files containing originall western blots for Figure 3I,indicating the relevant bands and treatments. copy/Source data Figure 3I uncropped western blots 1.pdf]

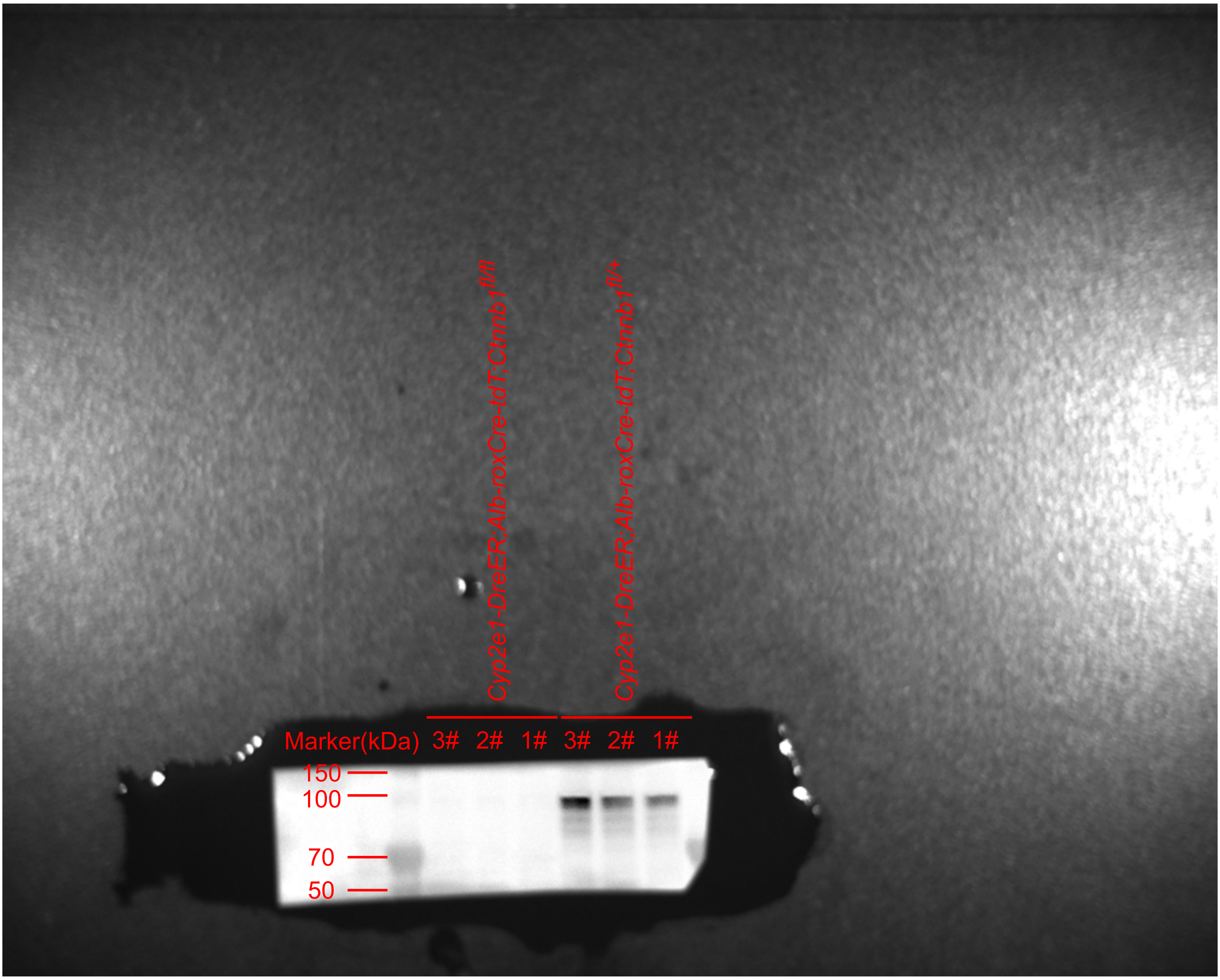

Cyp2e1-DreER;Alb-roxCre-tdT;Ctnnb1fl/fl
Cyp2e1-DreER;Alb-roxCre-tdT;Ctnnb1fl/+
Marker(kDa)
3#
2#
1#
3#
2#
1#
150
100
70
50
