# Supplementary material for: The robust, high-throughput, and temporally regulated roxCre and loxCre reporting systems for genetic modifications in vivo
Source: eLife. 2026 Apr 20;13:RP97717. doi: 10.7554/eLife.97717 (PMC13095210; doi:10.7554/eLife.97717)
Supplement: Figure 7—figure supplement 2—source data 2. [file elife-97717-fig7-figsupp2-data2.zip › Figure 7ΓÇö figure supplement 2ΓÇösource data 2/Source data Figure 7ΓÇö figure supplement 2_ Alb-roxCre7-GFP's the agarose gel electrophoresis results.pdf]

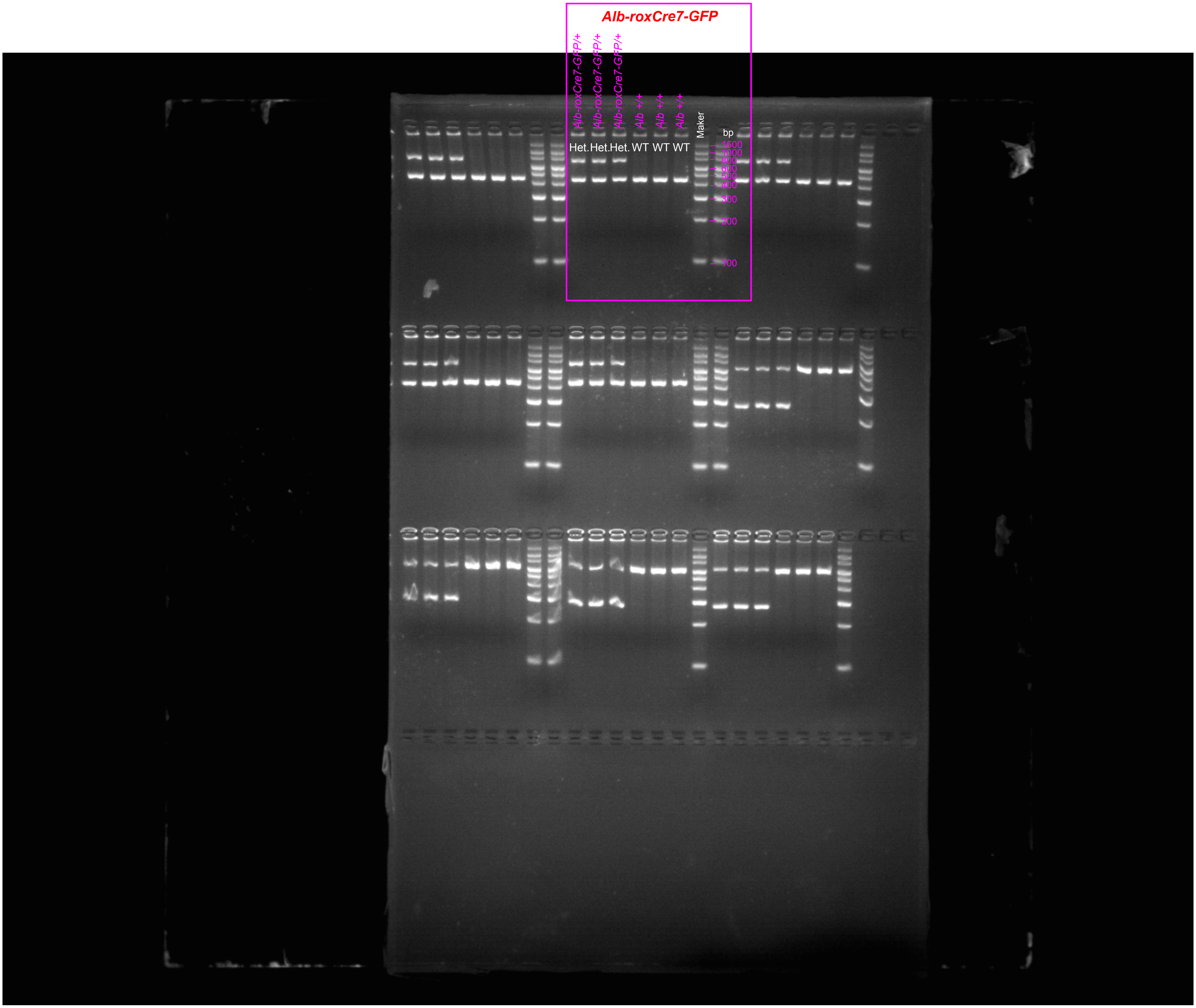

Alb-roxCre7-GFP
Alb-roxCre7-GFP/+
Alb-roxCre7-GFP/+
Alb-roxCre7-GFP/+
Alb +/+
Alb +/+
Alb +/+
Maker
bp
Het. Het. Het. WT WT WT
1500
1000
800
600
500
400
300
200
100
