# Supplementary material for: The robust, high-throughput, and temporally regulated roxCre and loxCre reporting systems for genetic modifications in vivo
Source: eLife. 2026 Apr 20;13:RP97717. doi: 10.7554/eLife.97717 (PMC13095210; doi:10.7554/eLife.97717)
Supplement: Supplementary file 1. — The subheadings in this document correspond to individual figures. The leftmost column indicates the lettered labels within each figure. The middle column provides descriptive annotations for the experimental groups. The right column specifies the detailed mouse genotypes for each respective group. [file elife-97717-supp1.docx]

**Mouse genotypes**

**Figure 1**

| **#** | **Information** | **Detailed genotype** |
| --- | --- | --- |
| C | The left part | *Rosa26-tdT* |
|  | The right part | *Rosa26-RSR-Cre;Rosa26-tdT* |
| E | No Tam control | *Rosa26-RSR-Cre2;Rosa26-GFP* |
| G | No Tam control | *Rosa26-R-reverseCre-R;Rosa26-tdT* |

**Figure 2**

| **#** | **Information** | **Detailed genotype** |
| --- | --- | --- |
| B | 1 | *Rosa26-DreER;Alb-roxCre1-tdT;Rosa26-Confetti* |
|  | 2 | *Rosa26-DreER;Alb-roxCre7-GFP;Rosa26-Confetti* |
|  | 3 | *Alb-CreER;Rosa26-Confetti* |
| E | 4 | *Rosa26-DreER;Cdh5-roxCre4-tdT;Rosa26-Confetti* |
|  | 5 | *Rosa26-DreER;Cdh5-roxCre10-GFP;Rosa26-Confetti* |
|  | 6 | *Cdh5-CreER;Rosa26-Confetti* |

**Figure 2—figure supplement 1**

| **#** | **Information** | **Detailed genotype** |
| --- | --- | --- |
| C | No Tam control | *Alb-roxCre1-tdT;Rosa26-GFP* |
| F | No Tam control | *Cdh5-roxCre4-tdT;Rosa26-GFP* |

**Figure 2—figure supplement 2**

| **#** | **Information** | **Detailed genotype** |
| --- | --- | --- |
| C | No Tam control | *Alb-roxCre7-GFP;Rosa26-tdT* |
| F | No Tam control | *Cdh5-roxCre10-GFP;Rosa26-tdT* |

**Figure 2—figure supplement 3**

| **#** | **Information** | **Detailed genotype** |
| --- | --- | --- |
| C | Left (no Tam);  right (Tam) | *Rosa26-DreER;Alb-roxCre1-tdT;Rosa26-GFP* |
| D | Left (no Tam);  right (Tam) | *Rosa26-DreER;Alb-roxCre7-GFP;Rosa26-tdT* |
| F and G | Left panel (pink panel) | *Rosa26-DreER;Alb-roxCre1-tdT;Rosa26-GFP* |
|  | Right panel (green panel) | *Rosa26-DreER;Alb-roxCre7-GFP;Rosa26-tdT* |

**Figure 2—figure supplement 4**

| **#** | **Information** | **Detailed genotype** |
| --- | --- | --- |
| C | Left panel | *Rosa26-DreER;Alb-roxCre1-tdT;Rosa26-Confetti* |
|  | Left—middle panel | *Rosa26-DreER;Cdh5-roxCre4-tdT;Rosa26-Confetti* |
|  | Right—middle panel | *Rosa26-DreER;Alb-roxCre7-GFP;**Rosa26-Confetti* |
|  | Right panel | *Rosa26-DreER;Cdh5-roxCre10-GFP;Rosa26-Confetti* |

**Figure 2—figure supplement 5**

| **#** | **Information** | **Detailed genotype** |
| --- | --- | --- |
| A | Left panel | *Rosa26-DreER;Cdh5-roxCre4-tdT;Rosa26-Confetti* |
|  | Right panel | *Rosa26-DreER;Cdh5-roxCre10-GFP;Rosa26-Confetti* |

**Figure 3**

| **#** | **Information** | **Detailed genotype** |
| --- | --- | --- |
| C, D, and I–L | The group marked with white square | *Cyp2e1-DreER;Alb-roxCre-tdT;Ctnnb1^flox/+^* |
|  | The group marked with pink square | *Cyp2e1-DreER;Alb-roxCre-tdT;Ctnnb1^flox/flox^* |
| F and G | Up panel | *Cyp2e1-DreER;Alb-roxCre-tdT;Ctnnb1^flox/+^* |
|  | Down panel | *Cyp2e1-DreER;Alb-roxCre-tdT;Ctnnb1^flox/flox^* |

**Figure 3—figure supplement 1**

| **#** | **Information** | **Detailed genotype** |
| --- | --- | --- |
| C–F |  | *Cyp2e1-DreER;Rosa26-RSR-tdT* |
| G | Left panel | *Cyp2e1-DreER;Rosa 26-RSR-tdT* |
|  | Right panel | *Cyp2e1-DreER;Alb-roxCre-tdT* |

**Figure 4**

| **#** | **Information** | **Detailed genotype** |
| --- | --- | --- |
| D | Left panel (Strategy 1) | *Rosa26-loxCre-tdT;Rosa26-tdT* |
|  | Right panel (Strategy 2) | *Rosa26-loxCre-tdT* |
| F | Left panel (Strategy 3) | *Cdh5-CreER;Rosa26-tdT* |
|  | Right panel (Strategy 4) | *Cdh5-CreER;Rosa26-loxCre-tdT* |

**Figure 4—figure supplement 1**

| **#** | **Information** | **Detailed genotype** |
| --- | --- | --- |
| A | Left panel | *Rosa26-loxCre-tdT* |
|  | Middle panel | *Rosa26-loxCre-tdT;Rosa26-tdT2* |
|  | Right panel | *Rosa26-loxCre-tdT* |
| B | Left panel | *Cdh5-CreER;Rosa26-tdT* |
|  | Right panel | *Cdh5-CreER;Rosa26-loxCre-tdT* |

**Figure 5**

| **#** | **Information** | **Detailed genotype** |
| --- | --- | --- |
| C and D | The group marked with white square | *Cdh5-CreER;Rosa26-Confetti* |
|  | The group marked with blue square | *Cdh5-CreER;Rosa26-loxCre-tdT;Rosa26-Confetti* |

**Figure 6**

| **#** | **Information** | **Detailed genotype** |
| --- | --- | --- |
|  | The group marked with white square | *Alb-CreER;Rosa26-tdT2;Rosa26-Confetti* |
|  | The group marked with flesh-toned red square | *Alb-CreER;iSuRe-Cre;Rosa26-Confetti* |
| C–E | The group marked with rosy flesh square | *Alb-CreER;Rosa26-loxCre-tdT;Rosa26-Confetti* |

**Figure 7**

| **#** | **Information** | **Detailed genotype** |
| --- | --- | --- |
| C | Up panel | *Alb-CreER;Rosa26-tdT2;Ctnnb1^flox/flox^* |
|  | Middle panel | *Alb-CreER;Rosa26-loxCre-tdT;Ctnnb1^flox/flox^* |
|  | Down panel | *Alb-CreER;Rosa26-loxCre-tdT;Ctnnb1^flox/+^* |
| D–G | The group marked with blue square | *Alb-CreER;Rosa26-tdT2;Ctnnb1^flox/flox^* |
|  | The group marked with red square | *Alb-CreER;**Rosa26-loxCre-tdT;Ctnnb1^flox/flox^* |
|  | The group marked with yellow square | *Alb-CreER;Rosa26-loxCre-tdT;Ctnnb1^flox/+^* |

**Figure 7—figure supplement 1**

| **#** | **Information** | **Detailed genotype** |
| --- | --- | --- |
| A |  | *Alb-CreER;Rosa26-loxCre-tdT* |
| B |  | *Alb-CreER;Rosa26-tdT;Ctnnb1^flox/flox^* |
| C | Group marked with circles | *Alb-CreER;Rosa26-tdT;Ctnnb1^flox/+^* |
|  | Group marked with triangles | *Alb-CreER;Rosa26-tdT;Ctnnb1^flox/flox^* |
| D |  | *Alb-CreER;Rosa26-tdT;Ctnnb1^flox/flox^* |
| E–G |  | *Alb-CreER;Rosa26-loxCre-tdT;Ctnnb1^flox/flox^* |
