# Supplementary material for: The robust, high-throughput, and temporally regulated roxCre and loxCre reporting systems for genetic modifications in vivo
Source: eLife. 2026 Apr 20;13:RP97717. doi: 10.7554/eLife.97717 (PMC13095210; doi:10.7554/eLife.97717)
Supplement: Supplementary file 2. — The leftmost column indicates the names of genes. The middle column provides related sequences. The right column describes the additional information on primers. [file elife-97717-supp2.docx]

**Oligos for *Mus musculus* genes**

| **Name of gene** | **Sequence** | **Additional information** |
| --- | --- | --- |
| *Alb-roxCre1-tdT*  (Mut) | TGGCAAACATACGCAAGGGA | PCR-forward primer |
|  | GCAAGCTTTCATTTATTCATCGCG | PCR-reverse primer |
| *Alb-roxCre1-tdT*  (WT) | TGGCAAACATACGCAAGGGA | PCR-forward primer |
|  | GTGGCAATGGTTCCTCTCTGC | PCR-reverse primer |
| *Cdh5-roxCre4-tdT*  (Mut) | TGCTTTGGGAGCTTGGACAT | PCR-forward primer |
|  | GTCGCAGATCTGCAAGCTTTCA | PCR-reverse primer |
| *Cdh5-roxCre4-tdT*  (WT) | TGCTTTGGGAGCTTGGACAT | PCR-forward primer |
|  | GGAAGGCTCCCAAAGCAGTG | PCR-reverse primer |
| *Alb-roxCre7-GFP*  (Mut) | TGGCAAACATACGCAAGGGA | PCR-forward primer |
|  | GCAAGCTTTCATTTATTCATCGCG | PCR-reverse primer |
| *Alb-roxCre7-GFP*  (WT) | TGGCAAACATACGCAAGGGA | PCR-forward primer |
|  | CCACCTAAGGGTTCTCAGATGC | PCR-reverse primer |
| *Cdh5-roxCre10-GFP*  (Mut) | CAGATCAGCTCCTCCACGAAGG | PCR-forward primer |
|  | GCAAGCTTTCATTTATTCATCGCG | PCR-reverse primer |
| *Cdh5-roxCre10-GFP*  (WT) | CAGATCAGCTCCTCCACGAAGG | PCR-forward primer |
|  | CCCCGCACCACCAATGATAAC | PCR-reverse primer |
| *Rosa26-loxCre-tdT*  (Mut) | CTGGTGCAAGCTGAACATAACTTCGT | PCR-forward primer |
|  | GGGAACCATTTCCTGTTGGATAACTTCG | PCR-reverse primer |
| *Rosa26-loxCre-tdT*  (WT) | GGCAGGGTCTCACTATGTATCTCTGC | PCR-forward primer |
|  | TTAAGCATGCTCTAACAGGCCTGG | PCR-reverse primer |
| *Cdh5-CreER*  (Mut) | GGCTACGAGGGCGCAGAGTCCAT | PCR-forward primer |
|  | CCGCCGCATAACCAGTGAAACAGC | PCR-reverse primer |
| *Cdh5-CreER*  (WT) | GAGGAGGGCGGTGGTGAGATGGA | PCR-forward primer |
|  | GAAGGGGCGCTGGGTTGAAGAGTC | PCR-reverse primer |
| *Cyp2e1-DreER*  (Mut) | GGGTCAGCCTTTGAAATGATAGC | PCR-forward primer |
|  | ATGAAGTTCTCAGCAGCCTCCTGG | PCR-reverse primer |
| *Cyp2e1-DreER*  (WT) | GGGTCAGCCTTTGAAATGATAGC | PCR-forward primer |
|  | GGTTGATACCAGCCAGGATACACC | PCR-reverse primer |
| *Ctnnb1* | CTATTACAACAGACTGCCTTC | qPCR-forward primer |
|  | AAGTGTAGGTCCTCATTATGT | qPCR-reverse primer |
| *Glul* | TGAACAAAGGCATCAAGCAAATG | qPCR-forward primer |
|  | CAGTCCAGGGTACGGGTCTT | qPCR-reverse primer |
| *Axin2* | GCTCCAGAAGATCACAAAGAGC | qPCR-forward primer |
|  | AGCTTTGAGCCTTCAGCATC | qPCR-reverse primer |
| *Cyp1a2* | ACATCTTTGGAGCTGGCTTT | qPCR-forward primer |
|  | GCTCCTCATGGATCTTCCTC | qPCR-reverse primer |
| *Cyp2e1* | TAACCAAGTTGGCAAAGCG | qPCR-forward primer |
|  | CGGCCAGAGAACTCATTCTT | qPCR-reverse primer |
| *Oat* | GGCTGTGGATCATGAGAATG | qPCR-forward primer |
|  | GCACTGCAGACACAGGGTAT | qPCR-reverse primer |
| *Tcf7* | AGCTTTCTCCACTCTACGAACA | qPCR-forward primer |
|  | AATCCAGAGAGATCGGGGGTC | qPCR-reverse primer |
| *Lect2* | TAGCAGGACCATGGGCTAAC | qPCR-forward primer |
|  | GCCCACTATCTTCCCAGTGA | qPCR-reverse primer |
| *Tbx3* | GTTCCCTGTCTGGAGTGGAT | qPCR-forward primer |
|  | GGTGAGGTAGGAAGGGATGA | qPCR-reverse primer |
| *Slc1a2* | ATGTCCACGACCATCATTGC | qPCR-forward primer |
|  | ACCTCGTCGTTCTTCTTCCC | qPCR-reverse primer |
| *Rhbg* | GTGTGGGCTTTACCTTCCTCG | qPCR-forward primer |
|  | CGCAGAAGTCAGCGTTGAT | qPCR-reverse primer |
